# Distinguishing Hemophagocytic Lymphohistiocytosis, Immune Reconstitution Inflammatory Syndrome, and HIV-Associated Immune Thrombocytopenic Purpura: A Challenging Case of Thrombocytopenia in AIDS

**DOI:** 10.7759/cureus.87255

**Published:** 2025-07-03

**Authors:** Mashaal Khan, Ali Z Ansari, Sean Lief, Rahul R Tirumalareddy, Sri Vallabh Reddy Gudigopuram, Axel B Lichtenberg, Sanim A Choudhury, Hiram A Gandara, Rashad Ali

**Affiliations:** 1 Department of Internal Medicine, The University of Tennessee Health Science Center, Memphis, USA; 2 Department of Pathology and Laboratory Medicine, William Carey University College of Osteopathic Medicine, Hattiesburg, USA; 3 Department of Internal Medicine, William Carey University College of Osteopathic Medicine, Hattiesburg, USA; 4 Department of Internal Medicine, Idaho College of Osteopathic Medicine, Boise, USA; 5 Department of Obstetrics and Gynecology, South Central Regional Medical Center, Laurel, USA

**Keywords:** aids, antiretroviral therapy, bone marrow suppression, cholangiopathy, hemophagocytic lymphohistiocytosis, hiv, immune reconstitution inflammatory syndrome, immune thrombocytopenic purpura, kaposi sarcoma, pancytopenia

## Abstract

Thrombocytopenia is a frequent hematologic complication in advanced HIV/AIDS, presenting significant diagnostic challenges due to overlapping etiologies. Hemophagocytic lymphohistiocytosis (HLH), immune reconstitution inflammatory syndrome (IRIS), and HIV-associated immune thrombocytopenic purpura (HIV-ITP) can all manifest with overlapping clinical features, which, despite their differing frequencies, may complicate diagnosis and management. We present a case of a 33-year-old male patient with advanced HIV/AIDS, metastatic Kaposi sarcoma, and inconsistent antiretroviral therapy (ART) adherence, who developed progressive thrombocytopenia, anemia, and pancytopenia. Initial workup, including an elevated HScore, suggested a possible HLH diagnosis; however, further immunologic testing did not meet HLH criteria. In the absence of definitive findings, HIV-ITP was identified as the most likely etiology. This case highlights the importance of a systematic, exclusion-based diagnostic approach in evaluating thrombocytopenia in immunocompromised patients, particularly when multiple concurrent conditions obscure the clinical picture.

## Introduction

Thrombocytopenia is a common and clinically significant complication in patients with HIV/AIDS, often arising from advanced immunosuppression, opportunistic infections, or malignancies. HIV-associated immune thrombocytopenic purpura (HIV-ITP) is a multifactorial disorder driven by immune-mediated platelet destruction, impaired megakaryocyte function, splenic sequestration, and bone marrow suppression [1]. However, diagnosing HIV-ITP remains challenging due to its overlapping clinical features with other causes of thrombocytopenia. A comprehensive diagnostic evaluation is crucial to exclude alternative etiologies, particularly in cases complicated by concurrent conditions such as hemophagocytic lymphohistiocytosis (HLH) or immune reconstitution inflammatory syndrome (IRIS).

HLH is a severe hyperinflammatory syndrome that can affect immunocompromised individuals, including those with HIV/AIDS. Its hallmark features-pancytopenia, hepatosplenomegaly, hyperferritinemia, and elevated triglycerides-closely resemble those of HIV-ITP, complicating the diagnostic process [2]. The diagnosis of HLH relies on specific criteria, including hemophagocytosis on bone marrow biopsy, elevated ferritin and triglyceride levels, and other laboratory markers, though these findings may not always be present in HIV/AIDS patients [2,3]. Tools like the HScore, a validated scoring system used to estimate the probability of HLH, can aid in diagnosis but may have limitations in HIV-positive populations [4,5]. HLH is frequently triggered by viral infections such as Epstein-Barr virus or cytomegalovirus, as well as malignancies like Kaposi sarcoma [5]. The significant overlap of clinical and laboratory findings makes distinguishing HLH from HIV-ITP particularly challenging, especially in advanced HIV, where both conditions may coexist.

Another critical consideration in HIV/AIDS patients is IRIS, which can emerge following the initiation of antiretroviral therapy (ART). IRIS occurs when immune recovery leads to an exaggerated inflammatory response against previously undiagnosed or subclinical opportunistic infections or malignancies. This immune activation can cause a paradoxical worsening of symptoms, including hematologic abnormalities such as thrombocytopenia, further complicating the diagnostic workup. Given its shared clinical features with HLH and HIV-ITP, IRIS can be difficult to distinguish from these conditions, particularly in severely immunocompromised individuals with multiple overlapping health issues. The timing of ART initiation and the patient’s immune response are crucial factors in recognizing IRIS as a potential contributor to thrombocytopenia [6].

Kaposi sarcoma, a common HIV-associated malignancy, is another important factor in the differential diagnosis of thrombocytopenia. It can contribute to platelet depletion through bone marrow suppression, direct infiltration of malignant cells, or splenic sequestration. The diagnostic challenge is further compounded when Kaposi sarcoma presents with systemic manifestations, such as pulmonary or gastrointestinal involvement, which can mimic opportunistic infections like *Pneumocystis jirovecii* pneumonia or *Mycobacterium avium* complex. Given this complexity, a thorough diagnostic evaluation including laboratory testing, imaging, and biopsy is essential to accurately determine the etiology of thrombocytopenia in HIV/AIDS patients, particularly in the presence of multiple coexisting conditions [7].

## Case presentation

A 33-year-old male patient who was diagnosed with HIV six years prior and progressed to AIDS approximately three years ago presented to the emergency department with a five-day history of worsening symptoms, including productive cough, generalized malaise, chronic diarrhea, intermittent abdominal pain, and gastrointestinal bleeding manifested as melena and hematochezia. Additional symptoms included worsening fatigue, dyspnea on exertion, and intermittent fever. His medical history was notable for inconsistent adherence to ART with bictegravir, emtricitabine, and tenofovir alafenamide (Biktarvy), resulting in profound immunosuppression. A recent CD4 count was critically low at 21 cells/mm³. The patient also reported difficulty tolerating prophylactic sulfamethoxazole-trimethoprim (Bactrim) due to gastrointestinal side effects, increasing his susceptibility to opportunistic infections and HIV-related complications. He had experienced multiple ART interruptions over the prior three years, often due to transportation challenges, limited social support, and adverse effects from medications. His ART nonadherence had led to several hospitalizations for opportunistic infections and AIDS-related complications.

His medical history was further complicated by biopsy-confirmed Kaposi sarcoma, presenting as a large rectal mass with imaging evidence of possible pulmonary metastases, including widespread pulmonary nodules, lymphadenopathy, and splenomegaly. He had a history of recurrent opportunistic infections, including COVID-19 pneumonia, oral and esophageal candidiasis, and recurrent *Pneumocystis jirovecii* pneumonia, for which treatment was modified due to renal toxicity associated with Bactrim. Socially, he lived with his mother, who provided support, and denied smoking or drug use, with only occasional alcohol consumption. He had no history of incarceration or homelessness but reported inconsistent access to outpatient care due to logistical barriers. There was no significant family history of cancer or chronic disease.

On examination, the patient appeared cachectic and fatigued, with scleral icterus. He was tachycardic (115 beats per minute), tachypneic (32 breaths per minute), and hypoxic, requiring supplemental oxygen for an oxygen saturation of 80% on room air. His blood pressure was low but responsive to fluids. Lung auscultation revealed bilateral crackles and diminished breath sounds at the bases, suggesting multifocal pneumonia and/or pulmonary involvement of Kaposi sarcoma. Abdominal examination demonstrated diffuse tenderness with moderate ascites but no rebound tenderness, raising concerns for visceral involvement of Kaposi sarcoma. Petechiae were present on the lower extremities, likely secondary to thrombocytopenia, along with mild peripheral edema consistent with hypoalbuminemia and fluid retention.

Laboratory results on admission revealed severe pancytopenia, with a hemoglobin level of 5 g/dL, platelet count of approximately 30,000/µL despite transfusions, and leukopenia. According to previous medical records, these abnormalities had been present for several months, with worsening noted over the past few weeks prior to admission. Peripheral blood smear showed Howell-Jolly bodies, suggesting functional asplenia, though no hemophagocytosis was observed. Immunologic studies revealed a CD4 count of 10 cells/mm³ and an HIV viral load of 190 copies/mL, consistent with poor viral control. His HScore was 169, indicating a moderate probability of HLH, but normal natural killer cell function and interleukin-2 levels suggested an alternative etiology for his pancytopenia. Liver function tests were markedly abnormal, with an alkaline phosphatase level exceeding 1000 U/L and a total bilirubin of 14.3 mg/dL, suggestive of AIDS cholangiopathy. Renal function was impaired, with a peak creatinine of 2.6 mg/dL, indicating acute kidney injury likely due to a combination of volume depletion, nephrotoxic medications, and chronic illness. Table 1 provides a comprehensive summary of the laboratory findings associated with the patient's condition.

**Table 1:** Laboratory findings indicating severe pancytopenia, leukopenia, low CD4 count, elevated alkaline phosphatase, total bilirubin, and impaired renal function.

Test	Patient’s value	Reference range
Hemoglobin	5 g/dL	13.8–17.2 g/dL
Platelet count	30,000/µL	150,000–450,000/µL
CD4 count	10 cells/mm³	500–1,500 cells/mm³
HIV viral load	190 copies/mL	Undetectable (<20 copies/mL)
HScore	169	<169 (lower risk), ≥169 (higher risk)
Natural killer cell function	Normal	Normal
Interleukin-2	Normal	Normal
Alkaline phosphatase	>1000 U/L	44–147 U/L
Total bilirubin	14.3 mg/dL	0.1–1.2 mg/dL
Creatinine	2.6 mg/dL	0.6–1.3 mg/dL

**Figure 1:**
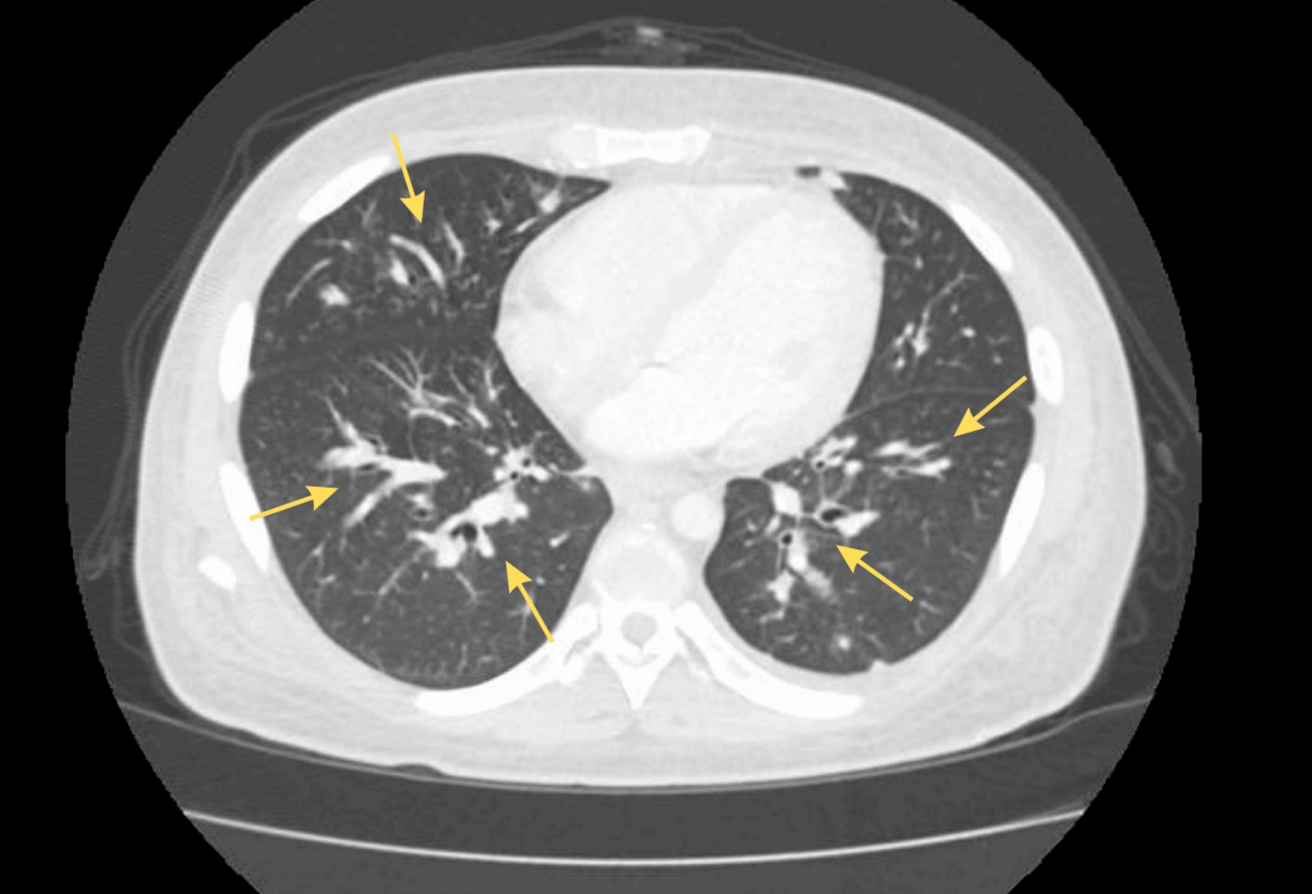
CT scan showing diffuse bilateral lung opacities with multiple nodular infiltrates (yellow arrows). CT: Computed tomography

Imaging studies delineated the extent of the patient’s disease. Chest X-ray and computed tomography (CT) scans revealed diffuse bilateral lung opacities with multiple nodular infiltrates, consistent with pulmonary Kaposi sarcoma metastases (Figure 1). Bilateral pleural effusions with adjacent atelectasis were also present, suggesting multifocal pneumonia, potentially due to concurrent *Pneumocystis jirovecii* pneumonia.

**Figure 2:**
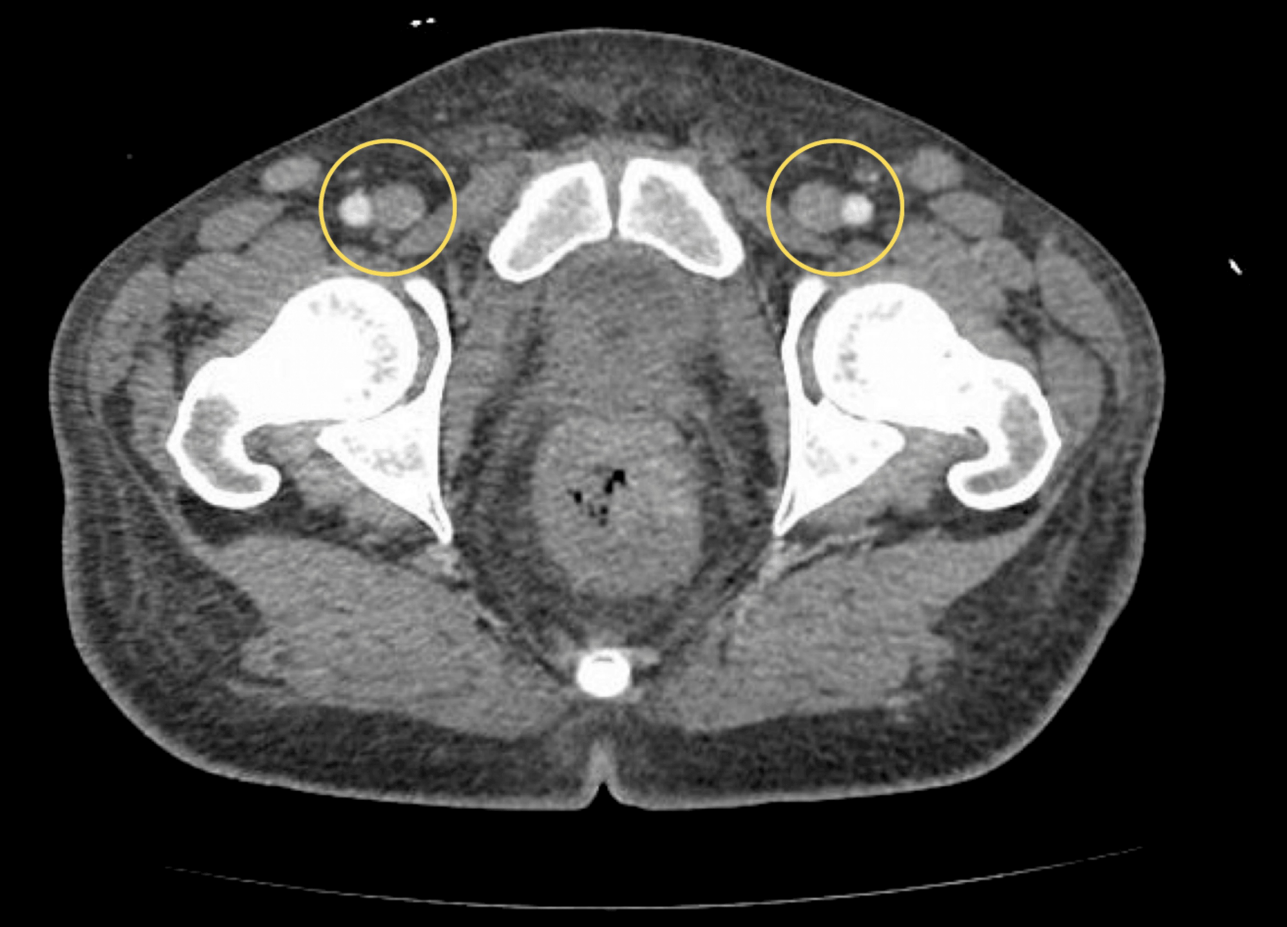
CT scan demonstrating extensive inguinal lymphadenopathy (yellow circles). CT: Computed tomography

Abdominal CT demonstrated extensive inguinal lymphadenopathy (Figure 2). Magnetic resonance imaging (MRI) findings indicated AIDS cholangiopathy, with biliary duct dilation contributing to hyperbilirubinemia and elevated alkaline phosphatase levels. Colonoscopy identified a highly vascular 12 cm rectal mass, confirmed on biopsy as Kaposi sarcoma, with additional lesions in the transverse colon, indicating multifocal gastrointestinal involvement.

During hospitalization, the patient received multidisciplinary care. Infectious disease specialists initiated empiric treatment for presumed *Pneumocystis jirovecii* pneumonia with atovaquone due to prior renal toxicity with Bactrim and started intravenous ganciclovir for cytomegalovirus viremia. Hematology managed his profound pancytopenia with transfusions to maintain hemoglobin above 7 g/dL and platelet counts above 10,000/µL. Although intravenous immunoglobulin (IVIG) and dexamethasone were administered for suspected HLH, the inconclusive diagnostic findings led to a cautious steroid taper. Due to his advanced disease and poor functional status, he was deemed ineligible for chemotherapy or surgical intervention for Kaposi sarcoma. Palliative radiation was considered for symptom management but deferred due to his unstable condition.

Nephrology managed his acute kidney injury conservatively, avoiding dialysis due to the bleeding risks associated with severe thrombocytopenia. A sodium bicarbonate infusion was used to address metabolic acidosis, and electrolyte abnormalities, including persistent hyperkalemia and hypoalbuminemia, were closely monitored. Despite aggressive supportive measures, the patient’s condition deteriorated, necessitating intensive care unit (ICU) transfer for mechanical ventilation due to respiratory failure and vasopressor support for hypotension. Given his rapid clinical decline, discussions were held with the patient’s family, and palliative care was consulted to prioritize comfort measures and quality of life in alignment with the patient’s goals of care.

## Discussion

Thrombocytopenia is a common hematologic complication in patients with HIV/AIDS, often arising from multiple contributing factors. In the context of HIV, it may result from immune-mediated platelet destruction, direct bone marrow suppression by the virus, splenic sequestration, or HIV-associated malignancies such as Kaposi sarcoma. In this case, the patient had a critically low CD4 count (21 cells/mm³) and persistent viral replication, indicating severe immunosuppression and emphasizing HIV’s role in thrombocytopenia through both direct and indirect effects on the bone marrow and immune system. Refractory thrombocytopenia in HIV warrants further evaluation, often including bone marrow biopsy, to differentiate underlying causes [8]. Given the multifactorial nature of thrombocytopenia in advanced HIV/AIDS, establishing a clear differential diagnosis is critical to guide appropriate therapy. While HIV-ITP is a well-recognized etiology, other conditions such as HLH, IRIS, drug-induced cytopenias, and malignancy-related bone marrow suppression can present with overlapping clinical features. Differentiating among these possibilities requires a systematic and exclusionary approach, as each carries distinct therapeutic implications. HLH may necessitate urgent immunosuppression, IRIS may call for ART modulation, HIV-ITP often responds to corticosteroids or IVIG, and infections or malignancies demand targeted antimicrobial or oncologic treatment. In the setting of profound immunosuppression, as in this patient, timely and accurate classification becomes especially important, as delayed or inappropriate interventions may lead to clinical deterioration. Despite transfusion support, the patient’s platelet count remained significantly low (30,000/µL), highlighting the contribution of HIV-ITP. However, thrombocytopenia in HIV/AIDS is not always solely attributable to the virus. In this case, while HIV-ITP was a consideration, the patient’s clinical and laboratory findings also raised suspicion for alternative conditions, particularly HLH and IRIS. The presence of severe pancytopenia, fever, and organ involvement-including hepatosplenomegaly, liver dysfunction, and ascites-necessitated a thorough evaluation to distinguish between these potential causes [1,2]. Precise epidemiologic data on the incidence of HLH and IRIS in patients with advanced HIV/AIDS remain limited, largely due to the rarity of these conditions and variability in diagnostic criteria and reporting. While HIV-ITP is recognized as a relatively common hematologic complication in this population, HLH and IRIS occur less frequently but carry significant clinical implications.

HLH is a life-threatening hyperinflammatory condition characterized by excessive activation of macrophages and T-cells, leading to widespread tissue damage, multi-organ failure, and significant hematologic abnormalities such as pancytopenia. It is commonly seen in immunocompromised patients, particularly those with HIV/AIDS, and is often triggered by infections (e.g., Epstein-Barr virus) or malignancies (e.g., Kaposi sarcoma). Diagnosis of HLH relies on two major criteria systems: the HScore and the HLH-2004 criteria. The HLH-2004 criteria require either a confirmed molecular diagnosis or the presence of at least 5 out of 8 clinical and laboratory findings, including fever, splenomegaly, cytopenia affecting at least two cell lines, hypertriglyceridemia and/or hypofibrinogenemia, ferritin ≥500 ng/mL, low or absent natural killer cell activity, hemophagocytosis in bone marrow or other tissues, and elevated soluble CD25 levels. In this case, no identifiable molecular diagnosis was present, and the patient met only 4 of the 8 criteria, falling short of a definitive HLH diagnosis. The patient’s elevated HScore (169) indicated a moderate probability of HLH, given the presence of pancytopenia, fever, liver dysfunction, and splenomegaly. However, additional investigations, including natural killer cell function testing and interleukin-2 levels, failed to meet the diagnostic threshold for HLH, emphasizing the limitations of scoring systems, particularly in the context of advanced HIV. Furthermore, the absence of hemophagocytosis on bone marrow biopsy and the lack of elevation in key HLH markers (e.g., ferritin, triglycerides) weakened the likelihood of HLH. This highlights the diagnostic challenge of differentiating HLH from other causes of thrombocytopenia and pancytopenia, particularly when HIV-related complications, such as HIV-ITP, present with overlapping clinical features [2,5,9].

IRIS is another critical differential diagnosis for thrombocytopenia in HIV/AIDS patients, particularly following the initiation or modification of ART. IRIS arises from an exaggerated inflammatory response triggered by immune recovery, leading to the unmasking or worsening of previously unrecognized subclinical opportunistic infections or malignancies. This phenomenon is most commonly observed in patients with advanced HIV, where ART initiation can paradoxically exacerbate symptoms due to immune reconstitution. In this case, the patient’s HIV was poorly controlled, and despite ongoing ART, his CD4 count remained extremely low (21 cells/mm³), indicating profound immunosuppression. The patient had experienced multiple ART interruptions over the prior three years due to transportation challenges, limited social support, and medication side effects, resulting in inconsistent adherence. There was no clear evidence of recent ART initiation or modification immediately preceding his hematologic decline, suggesting that classical IRIS triggered by ART changes was unlikely to be the primary cause. Nonetheless, his history of ART interruptions and recurrent opportunistic infections may have contributed to an IRIS-like inflammatory response exacerbating his thrombocytopenia and pancytopenia. A history of multiple opportunistic infections, including *Pneumocystis jirovecii* pneumonia and cytomegalovirus viremia, may have contributed to his clinical deterioration through IRIS [10]. His worsening pancytopenia could potentially be attributed to IRIS, as the inflammatory response to Kaposi sarcoma lesions and opportunistic infections may have triggered immune-mediated platelet destruction and bone marrow suppression. However, distinguishing IRIS from other causes of thrombocytopenia, such as HIV-ITP and HLH, remains a challenge due to overlapping clinical features. In this case, IRIS may have played a role in worsening thrombocytopenia and pancytopenia, but it was unlikely the primary cause, as the patient did not meet HLH diagnostic criteria and lacked clear evidence of an exacerbation of undiagnosed infections or malignancies following ART initiation. This case emphasizes the importance of considering IRIS in the differential diagnosis of hematologic abnormalities in HIV patients, particularly when ART is introduced or modified, while also recognizing the necessity of differentiating it from conditions like HLH and HIV-ITP [1,6,10].

Kaposi sarcoma is a vascular tumor that can contribute to thrombocytopenia through multiple mechanisms, including bone marrow suppression, splenic sequestration, and direct infiltration of malignant cells into the bone marrow [1,7]. In this case, Castleman’s disease, which is strongly associated with HIV and human herpesvirus 8 (HHV-8), should also be considered in the differential diagnosis, as its hallmark features, including thrombocytopenia, splenomegaly, and lymphadenopathy, overlap with the patient’s presentation. A lymph node biopsy would be valuable in further evaluating this possibility [11]. This patient had a known diagnosis of Kaposi sarcoma with gastrointestinal involvement and pulmonary metastasis. Biopsy-confirmed rectal and colonic lesions may have contributed to pancytopenia, likely due to vascular entrapment and splenic sequestration [7]. The role of Kaposi sarcoma in thrombocytopenia is often overlooked, particularly in patients with concurrent acute infections or inflammatory conditions [12,13]. In this case, the presence of Kaposi sarcoma, alongside the patient’s history of opportunistic infections, further complicated the diagnostic process, highlighting the need for a thorough evaluation to differentiate between malignancy-related cytopenias and other HIV-associated hematologic abnormalities.

Recent advances in HIV treatment have introduced long-acting injectable antiretroviral therapies (LA-ART), which have shown promise in improving adherence among patients facing challenges with daily oral regimens. LA-ART formulations, administered monthly or bimonthly, can reduce the burden of pill fatigue, minimize the impact of gastrointestinal side effects, and overcome barriers such as transportation or unstable living situations [14]. For patients like ours, who struggled with inconsistent ART adherence due to social and medication-related factors, LA-ART may represent a valuable alternative to enhance viral suppression and reduce the risk of disease progression. While not available or utilized in this case, incorporating LA-ART into treatment strategies for similar patients may improve outcomes and warrants further consideration in clinical practice.

## Conclusions

This case highlights the complexities involved in managing advanced HIV/AIDS, particularly when factors such as nonadherence to ART, severe immunosuppression, and multiple coexisting conditions are present. The patient's clinical profile, which included HIV-ITP, Kaposi sarcoma, AIDS cholangiopathy, opportunistic infections, and potential IRIS, required a multifaceted approach. Importantly, the exclusion of HLH and IRIS allowed the clinical team to avoid initiating potentially harmful treatments such as immunosuppressive therapy or ART modification, which could have further compromised the patient’s condition. Instead, targeted management focused on supportive care, platelet transfusions, infection control, and oncologic evaluation. This approach emphasized symptom management, exclusionary diagnostics, and interdisciplinary collaboration. There is a critical need for robust support systems to improve ART adherence and for early intervention in immunocompromised HIV patients to prevent disease progression. Improving the quality of life for individuals living with HIV requires addressing both medical and psychosocial needs. This includes providing stable access to ART, minimizing medication side effects, ensuring transportation to appointments, and offering mental health services, housing assistance, and substance use counseling when appropriate. Strengthening these social determinants of health can reduce barriers to care, improve treatment adherence, and ultimately enhance clinical outcomes and patient well-being. Ultimately, this case illustrates the value of a comprehensive, patient-centered approach in managing late-stage HIV/AIDS, particularly in contexts where advanced complications and a poor prognosis limit therapeutic options.
